# Vigo’s Plaster in Small-Pox

**Published:** 1849-06

**Authors:** 


*Vigo's Plaster in Small-Pox.*—The question of whether the action of Vigo's plaster in preventing and arresting the development and suppuration of small-pox pustules is specific, or possessed in common by other greases, is thus resolved by M. Champouillon. In 108 cases of variola, the subjects varying in age from twenty-one to twenty-eight years, the emplaster Vigo was applied to the face; the surface of the neck was covered at the same time with a layer of hog's lard, olive oil, or diachylon. In only four instances was the variolic eruption aborted in this latter situation, whilst it was developed upon the face in but eleven cases.—*Ib.*

